# Distinguishing between yield advances and yield plateaus in historical crop production trends

**DOI:** 10.1038/ncomms3918

**Published:** 2013-12-17

**Authors:** Patricio Grassini, Kent M. Eskridge, Kenneth G. Cassman

**Affiliations:** 1 Department of Agronomy and Horticulture, University of Nebraska–Lincoln, PO Box 830915, Lincoln, Nebraska 68583-0915, USA; 2 Department of Statistics, University of Nebraska–Lincoln, Lincoln, Nebraska 68583-0963, USA

## Abstract

Food security and land required for food production largely depend on rate of yield gain of major cereal crops. Previous projections of food security are often more optimistic than what historical yield trends would support. Many econometric projections of future food production assume compound rates of yield gain, which are not consistent with historical yield trends. Here we provide a framework to characterize past yield trends and show that linear trajectories adequately describe past yield trends, which means the relative rate of gain decreases over time. Furthermore, there is evidence of yield plateaus or abrupt decreases in rate of yield gain, including rice in eastern Asia and wheat in northwest Europe, which account for 31% of total global rice, wheat and maize production. Estimating future food production capacity would benefit from an analysis of past crop yield trends based on a robust statistical analysis framework that evaluates historical yield trajectories and plateaus.

## Abstract (web summary)

Food security and the conservation of natural ecosystems largely rely on the increase in crop yields. Here, the authors examine global crop yield trends since 1960, and establish a robust statistical framework for estimating historical trajectories and identifying yield plateaus.

## Graphical abstract

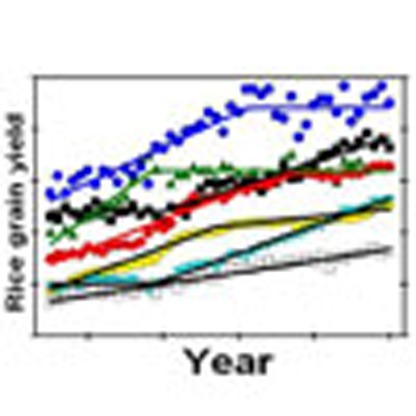

The first decade of the new millennium saw an abrupt reversal of long-term trends in declining grain prices since the onset of the green revolution in the mid-1960s, and an increase in expansion of land area used for crop production. Whether this recent expansion in crop area is transitory or permanent will depend in large part on trajectories in grain and oilseed prices, which in turn depend on trends in crop yields. Estimating these trends with a high degree of confidence is therefore essential to inform development of appropriate agricultural policies and priorities for agricultural research to ensure future food security and minimize conversion of carbon-rich and biodiverse natural ecosystems to cropland, which contributes substantially to anthropogenic greenhouse gas emissions and climate forcing^1^,^2^,^3^. In fact, agricultural production, including indirect emissions associated with land-use change, accounts for 15–25% of the total anthropogenic greenhouse gas emissions^4^,^5^. Hence, a critical question is whether current yield trajectories are adequate to achieve the needed production increases on existing farmland because econometric projections of grain prices and land use change are highly sensitive to underpinning assumptions about future crop yield increases.

Here, by applying a robust, statistical framework to evaluate historical yield trends of major crop-producing countries since 1965, we find that all trends can be described by linear functions with or without an abrupt decrease in rate of gain or an upper yield plateau. We conclude that estimates of future crop production and land use must consider both historical yield trends and biophysical yield ceilings to improve forecasting capability.

## Results

### Recent trends in land use for crop production

**Figure 1:**
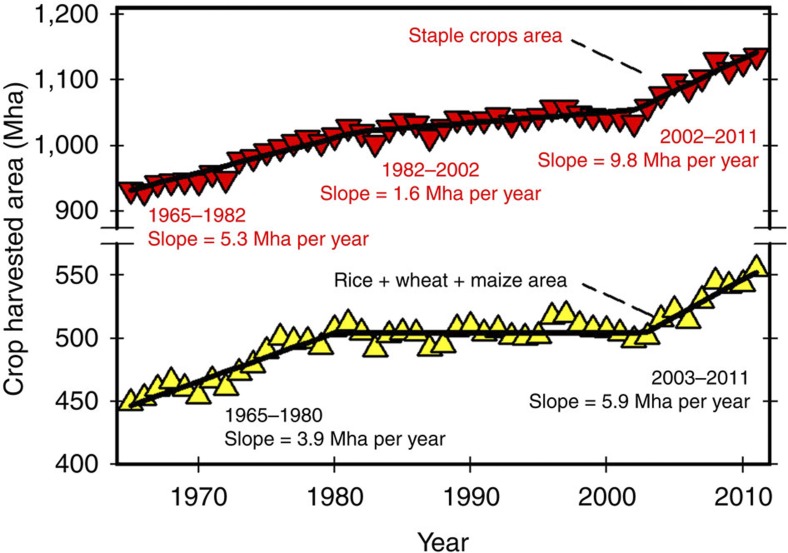
Trends in total harvested area of staple crops and three major cereal crops. Staple crops include cereal, oil, sugar, pulses, fibre, tuber plus root crops. The three major cereal crops are rice, wheat and maize. Slopes of the fitted trilinear models are shown when significant (Student’s *t*-test; *P*<0.01, *n*=47 years of yield data).

At a global level, changes in land use for crop production have been driven in large part by increases in land area devoted to the three major cereals (rice, wheat and maize). During the first 16 years of the green revolution, for example, expansion of area for the major cereals represented >70% of land use increase for all crops, followed by two decades in which both total crop area and area in major cereals remained relatively constant (Fig. 1 and Supplementary Table 1). This period of stability came to an abrupt end in 2002. Since then, crop production area has increased at nearly ten million hectares per year and 60% of this expansion is due to increased production of the major cereals. An additional 25% is attributable to expansion of soybean area. Nearly all of the increased crop area since 2002 has occurred in South America, Asia and Africa (Supplementary Table S1).

### Previous analyses of historical crop yield trends

**Figure 2:**
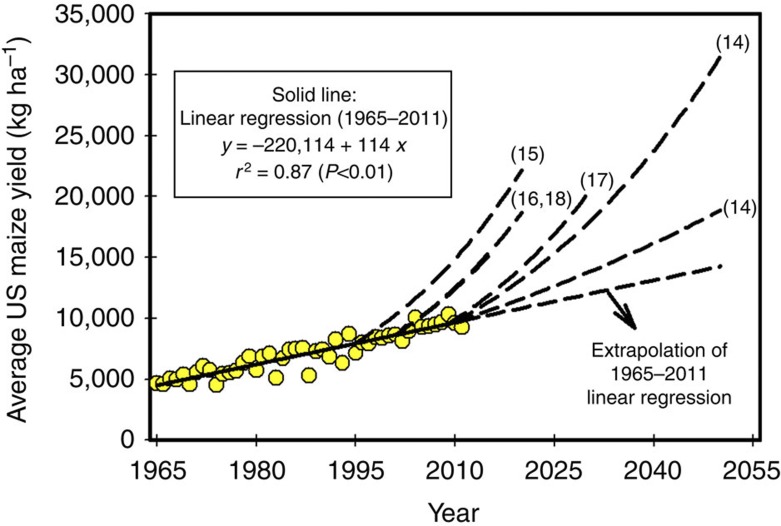
Historical trend in average US maize yield and reported projected trajectories based on compound rates of yield gain. Historical trend (1965–2011, *n*=47 years of yield data) is indicated with the solid line and yellow data points, and associated linear-regression equation, coefficient of determination (*r*^2^) and Student’s *t*-test *P*-value are shown. Trajectories reported in publications that evaluated future food production potential based on these projected yield trajectories are indicated with the dashed lines. Numbers associated with each trajectory indicate the reference in which this exponential rate was used. The trajectory based on extrapolation of the 1965–2011 linear regression is also shown.

Global rates of yield increase have been decidedly linear for most major cereal crop species since the start of the green revolution in the 1960s^6^,^7^. Some projections of global food security assume that these linear rates of increase will continue unchanged during the next 40 years^8^,^9^,^10^,^11^. Other projections, including several based on computational partial equilibrium models that evaluate both food demand and supply, assume compound annual rates of yield increase without recognizing biophysical limits to crop yields^12^,^13^,^14^,^15^,^16^,^17^,^18^ (Fig. 2 and Supplementary Table S2). In a compound rate model, annual yield gain represents a constant proportion of the current yield and thus the magnitude of absolute gain increases each year. Although exponential increase in crop yields can occur over short time periods of one or two decades, such growth rates are not feasible over the long term because average farm yields eventually approach a yield potential ceiling determined by biophysical limits on crop growth rates and yield^19^,^20^,^21^,^22^,^23^. For irrigated crops, yield potential is defined as the yield of a crop cultivar when growth is only limited by solar radiation, temperature and carbon dioxide supply from the atmosphere, and also by the amount and distribution of precipitation in rainfed cropping systems^23^,^24^.

To account for the expected deceleration in crop yields as they approach their yield potential ceiling, several recent projections of food security have considered a declining compound rate over time, which aims to mimic the shape of an asymptotic trend^25^,^26^,^27^. Another recent study suggests that yields have stagnated in a large portion of the world’s crop production area, and that current yield trends are not sufficient to meet the demand on existing farmland^28^. However, this study is based on yield trend estimates and their pattern over time using gridded, historical crop yield data derived from average yields reported at coarser levels of spatial resolution. In addition, many of the highlighted yield trends came from regions with negligible crop production (e.g., maize in Moldova, soybean in Congo, rice in Greece). The statistical analysis was limited to only three models, including the cubic model to detect yield plateaus—which has no biophysical justification—and model selection was not robust because regression analysis assumptions were not tested. Only one recent study followed a robust, statistically sound method to describe yield trends, but that study was limited to one crop (wheat) and only two statistical models were evaluated^29^.

Exaggerated yield gain rates, sometimes justified by unverifiable ‘record’ crop yields, have been used to support optimistic projections of grain production^12^,^13^,^14^,^15^,^16^,^17^,^18^ (Fig. 2 and Supplementary Table S2). For instance, a widely publicized goal for average US maize yield of 20 Mg ha^−1^ by 2030 would require an annual yield gain of 506 kg ha^−1^ per year (or 3.6% per year compounded)^17^. This rate is four times greater than the rate of increase in US maize yield from 1965 to 2011 (114 kg ha^−1^ per year). Another recent study predicted a 2.8% per year rate of gain in US maize yield (equivalent to 460 kg ha^−1^ per year as a linear rate) under the assumption that farmers would respond to the incentive of higher grain prices caused by increased global demand, partly as a result of the US bioethanol mandates^18^.

### Improved analytical framework for estimating historical yield trends

Trajectories in national average yields are driven by changes in crop management practices, crop genetic improvement through conventional breeding and genetic engineering, climate and interactions among these factors, under influence of surrounding social, economic and political environments^30^. Depending on country and time period, the mathematical form of historical yield trend for a specific crop in a given country can be linear, exponential, parabolic, linear plateau or flat. Statistical trend analysis is the only objective method to determine the mathematical model that best fits observed data. And although food security projections and yield gain rates of major cereal crops are extremely sensitive to mathematical form, rigorous statistical trend analysis to identify the most appropriate statistical model (hereafter called ‘best-fit model’) have not been used to inform previous projections of global food security as published in the papers cited herein.

**Figure 3:**
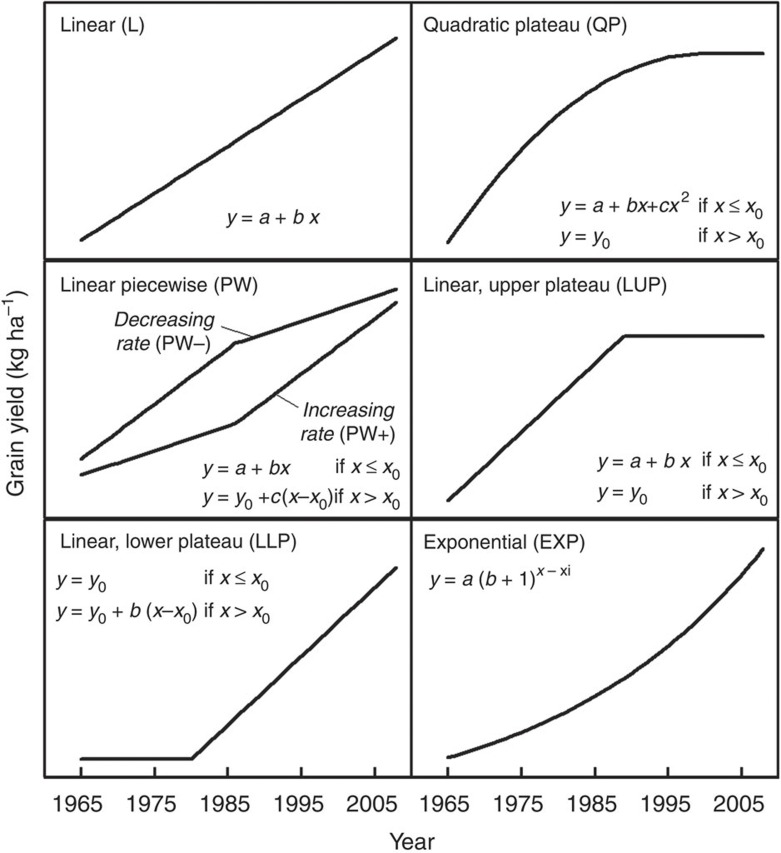
**Six statistical models** **evaluated for their performance to fit observed crop yield trends.** For piecewise and linear-plateau models, *x*_0_ is the breakpoint year and *y*_0_ is the yield value of the upper (LUP) or lower plateau (LLP), or in the breakpoint year of the PW model.

**Figure 4:**
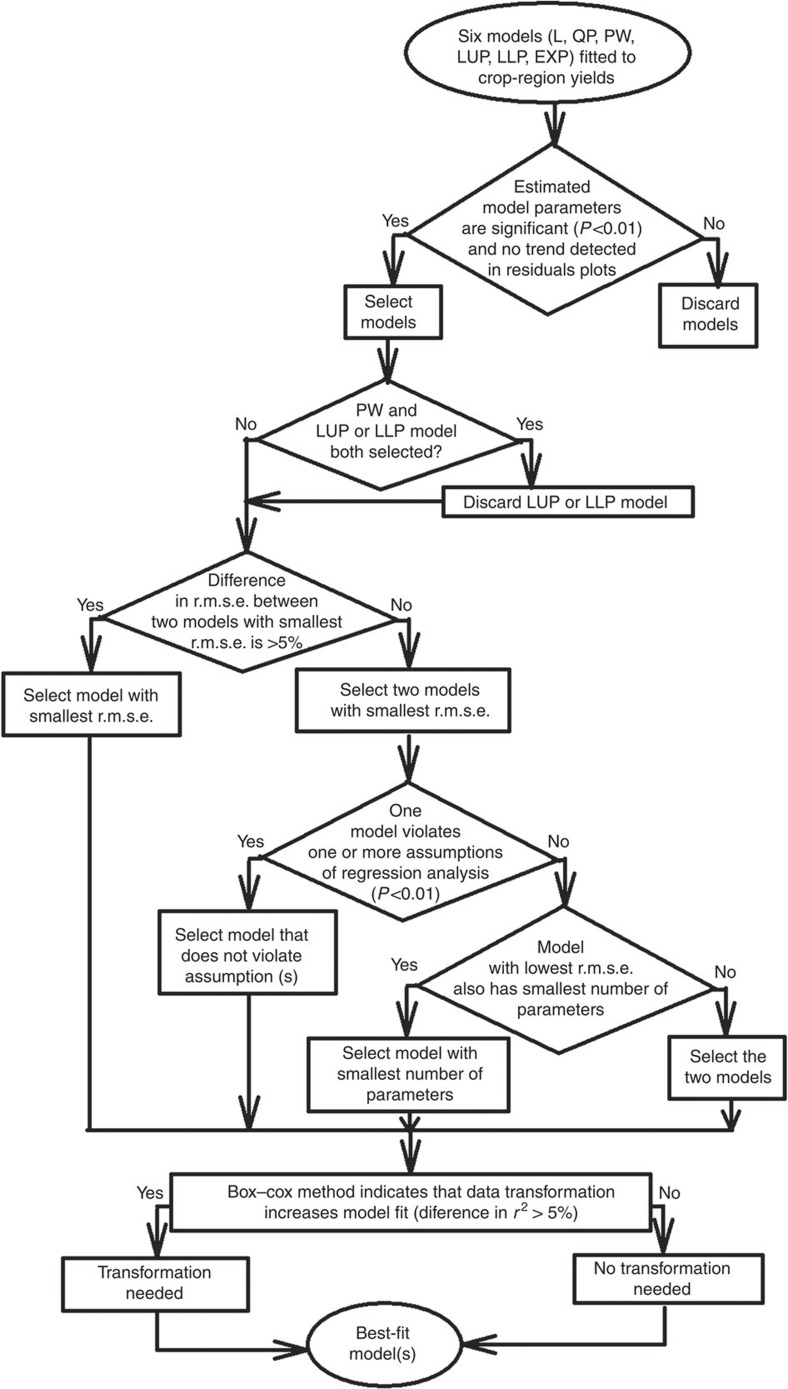
Decision tree used to identify best-fit models for each crop–country or –region case. Note the description of statistical tests and detailed explanation of the cases exhibiting significant serial correlation, as detected with the Durbin–Watson test and the residual plots, in the Methods section. r.m.s.e., root mean square error; r^2^, coefficient of determination.

Here we develop a framework for statistical trend analysis of historical crop yields and use it to analyse yield trends of the major cereals since the start of the green revolution in 36 countries and regions, which together account for 84, 56 and 71% of global rice, wheat and maize production, respectively (Supplementary Table S3). These three crops are the focus of our analysis because rice, wheat and maize together account for ~85% of global cereal production and contribute a majority of human calories eaten directly as staple foods or indirectly through consumption of livestock fed with grain. Six statistical models, widely used in the literature for describing time series trends in crop yields, provided the basis for a comprehensive analysis of historical crop yield trends (Fig. 3). Testing all six models based on statistically sound criteria provided an objective approach for identifying the most appropriate shape of historical yield trend (Fig. 4). From this analysis, we estimate the proportion of global grain production from countries and regions in which yields are flat, rising, declining or plateauing, consider the most plausible explanations for cases of accelerating or plateauing yields and posit implications of these findings for studies of future food production capacity and land area required to produce it.

### Rate of increase in cereal crop yield is generally linear

**Figure 5:**
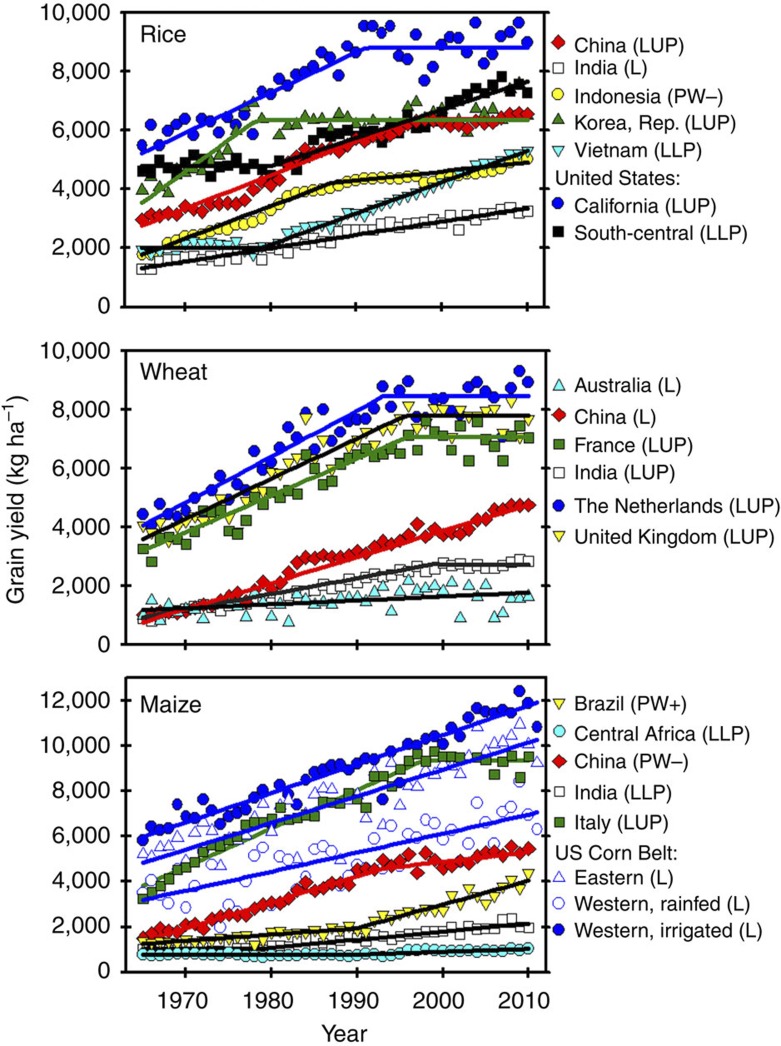
Trends in grain yield of the three major cereal crops for selected regions since the start of the green revolution in the 1960s. Fitted model for each crop–region case is indicated in parenthesis. L, linear; QP, quadratic plateau; PW, piecewise with (+) increasing or (−) decreasing rate after breakpoint year; LUP or LLP, linear with upper or lower plateau; EXP, compound exponential.

Best-fit trends for selected crop–country and –region cases are shown in Fig. 5. Linear models, with or without a discontinuous breakpoint, adequately described all of the yield trends (Table 1, Fig. 5 and Supplementary Table S4). In 10 of 36 cases, a linear rate of gain throughout the entire time series provided the best fit. Dividing the rate of gain by the trendline yield provides the relative rate of gain as a percentage of the estimated yield for a given year (Supplementary Table S5). For example, average rice yield in India was ~1,500 kg ha^−1^ in 1970, whereas the rate of gain was 45 kg ha^−1^ per year, which gives a relative rate of gain of 2.9%. By 2010, average rice yield had risen to ~3,300 kg ha^−1^, which means the relative rate of gain had fallen to 1.3%. Thus, in 28% of the cases evaluated, rate of increase remains constant such that relative rates of gain decline throughout the entire time series (Supplementary Table S5).

Decreasing absolute rates of gain were observed for rice in Indonesia and maize in China, where yield gain during recent decades (31 and 42 kg ha^−1^ per year, respectively) are much slower than rates at the beginning of the time series (110 and 115 kg ha^−1^ per year, respectively). In contrast, abrupt, transitory increases in both absolute and relative rates of yield gain were observed in 11 cases (Supplementary Table S5). These cases corresponded to crops and countries where little yield increase occurred at the beginning of the time series and average yield remained low for a long time period, such as rice in Vietnam and maize in Brazil (Fig. 5). For rice in Vietnam, socioeconomic stabilization following the Vietnam War, introduction of modern indica rice varieties and increased use of fertilizer supported an abrupt upward yield trend beginning in 1979. In Brazil, the development of improved management practices for acid, infertile soils in the Cerrado and the introduction of hybrid seed led to a rapid rise in maize yields after 1990. However, even in these cases, subsequent yield gain after the abrupt upturn was remarkably linear and therefore relative rates of gain decline (Supplementary Table S5).

### Strong evidence of yield plateaus in some of the world’s most intensive cropping systems

A major concern is the observation that yields in some major cereal-producing regions have not increased for long periods of time following an earlier period of steady linear increase, hereafter called ‘upper yield plateaus’ (Table 1, Fig. 5 and Supplementary Table S4). Upper yield plateaus were observed in 14 of the 36 cases. Moreover, there was no case of a return to the previous rising yield trend after a statistically significant upper yield plateau occurred. Taken together, cases with statistically significant upper yield plateaus represent 33% of global rice and 27% of global wheat production (Table 2). Given the importance of these intensive systems to global food supply, identification of underpinning causes for yield plateaus in these systems is fundamental for estimating future food production capacity. A hypothesis that can explain the occurrence of yield plateaus is that average farm yields approach a biophysical yield ceiling for the crop in question, which is determined by its yield potential in the regions where the crop is produced^19^,^20^,^21^. This seems to be the case in high-yield systems for rice in East Asia (China, Republic of Korea and Japan), wheat in Northwest Europe (United Kingdom, France, Germany, The Netherlands, Denmark) and India, and maize in South Europe (Italy and France). Other factors may also contribute to observed yield plateaus, including cyclical weather patterns, land degradation, shift in the location of production area to regions with poorer soils and climate, policies on the use of fertilizers and pesticides, and insufficient or poorly oriented investment in agricultural research and development (R&D)^27^,^29^,^31^.

At issue is how many years of plateau are needed before the trend becomes statistically significant. Analysis of cases in this study indicates that 4–18 years of ‘flat’ yields are needed to detect a statistically significant yield plateau, depending on the degree of year-to-year variability in yield along the entire time series (Supplementary Table S6). In favourable irrigated or rainfed environments where yield variability due to weather and water supply is small, ~8 years are needed to identify a significant yield plateau (for example, irrigated rice in China and California, rainfed wheat in Northwestern Europe and maize in South Europe). Longer time series (13–18 years) are needed to identify yield plateaus in harsher rainfed cropping systems due to the high year-to-year variability in yield, such as for wheat in the southern US Great Plains where it appears there has been little improvement in yields of recent varietal releases^22^.

Length of the ascending linear phase is also important. For example, a recent study of wheat yield trends in three regions in The Netherlands reports that average farm yield continues to rise at a linear rate^32^, in contrast to our analysis (Table 1 and Fig. 5). The reason for this different conclusion is that the Dutch study uses a shorter time series, which begins in 1979, and thus has only 15 years of ascending linear phase, based on the breakpoint year (1993) found in the present study. In our analysis, the time series begins in 1965 and thus has 29 years to establish the linear phase.

In contrast to plateaus in high-yield systems, yield stagnation at low-yield levels or very low rates of yield gain are observed in countries or regions where farmers lack access to agricultural inputs, infrastructure, capital, markets and extension services, such as for maize in Africa (Supplementary Table S4). These regions exhibit the highest potential for intensification because substantial increases in crop production can be achieved without expansion of current cropland area, through modest increases in current yield gain rates. For example, Africa accounts for 15% of global maize harvested area but produces <5% of current global maize. With current rates of yield gains of 0, 13 and 29 kg ha^−1^ per year (East, Central and West Africa, respectively), total maize production in Sub-Saharan Africa will increase only by 9% in the next 10 years which, in turn, will put pressure on expanding cropland area as observed during the last decade (Fig. 1 and Supplementary Table S1). In contrast, if current yield gains could be increased to a modest rate of 80 kg ha^−1^ per year, similar to the rates observed for maize in other harsh environments such as the western US Corn Belt, total maize production in Sub-Saharan Africa would increase by 53% in the next 10 years, helping to decrease pressures to expand cropland area and food imports.

### No support exists for use of compound, exponential rates of yield increase to project yield trends

**Figure 6:**
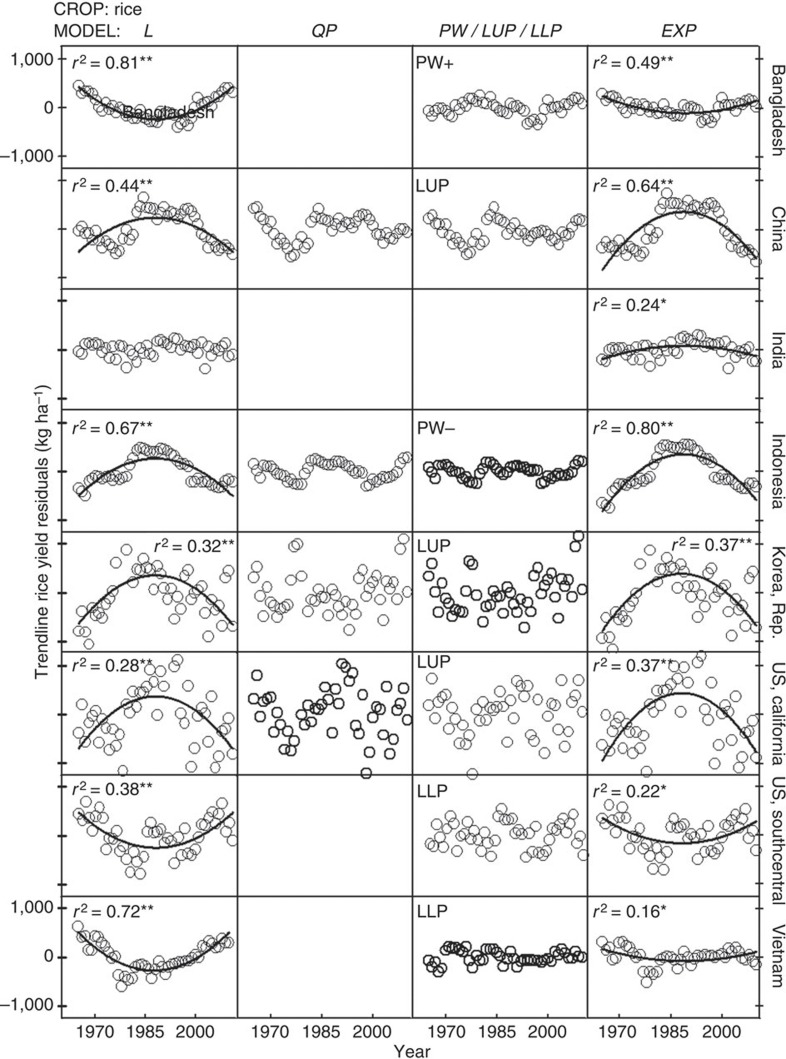
Trendline residuals for rice yield plotted against year for selected regions. Selected regions are indicated in the right axis labels. Only one residual plot is shown for either the piecewise (PW+, PW−) or linear plateau with upper/lower plateau (LUP/LLP) because these models are mutually exclusive. The *y* axis scale is the same across all panels and is shown only in the upper and bottom panels. Asterisks indicate significance at *F*-test *P<0.01 or **P<0.001 of the fitted regression line to the residuals over time (*n*=46 years of yield data, except for USA (*n*=47)). L, linear; QP, quadratic plateau; PW, piecewise with (+) increasing or (−) decreasing rate after breakpoint year; LUP/LLP, linear with upper/lower plateau; EXP, compound exponential.

**Figure 7:**
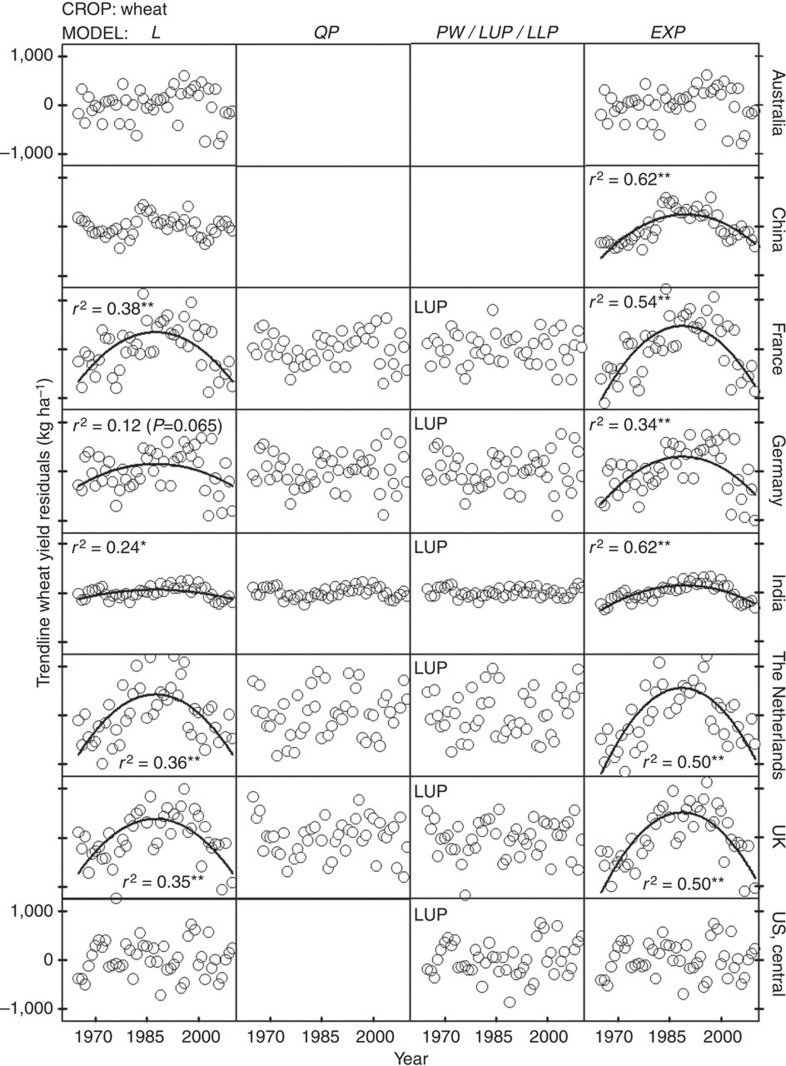
Trendline residuals for wheat yield plotted against year for selected regions. Selected regions are indicated in the right axis labels. Only one residual plot is shown for either the piecewise (PW+, PW−) or linear plateau with upper/lower plateau (LUP/LLP) because these models are mutually exclusive. The *y* axis scale is the same across all panels and is shown only in the upper and bottom panels. Asterisks indicate significance at *F*-test *P<0.01 or **P<0.001 of the fitted regression line to the residuals over time (*n*=46 years of yield data, except for USA (*n*=47)). L, linear; QP, quadratic plateau; PW, piecewise with (+) increasing or (−) decreasing rate after breakpoint year; LUP/LLP, linear with upper/lower plateau; EXP, compound exponential.

**Figure 8:**
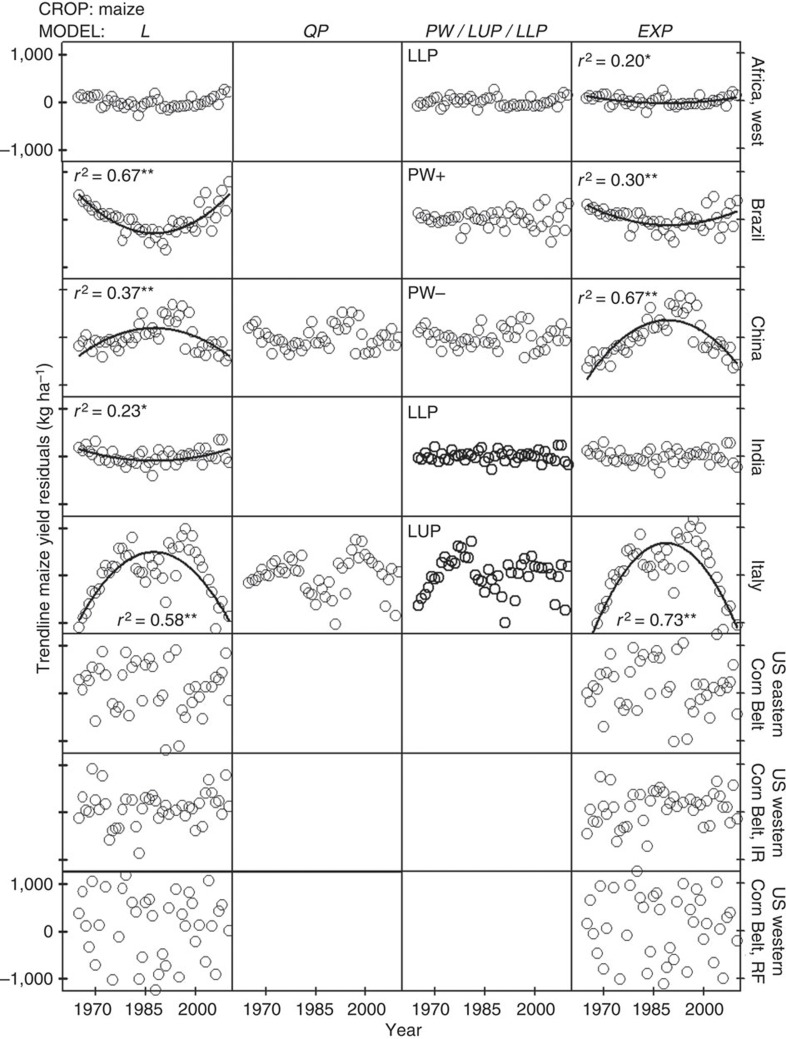
Trendline residuals for maize yield plotted against year for selected regions. Selected regions are indicated in the right axis labels. Only one residual plot is shown for either the piecewise (PW+, PW−) or linear plateau with upper/lower plateau (LUP/LLP) because these models are mutually exclusive. The *y* axis scale is the same across all panels and is shown only in the upper and bottom panels. Asterisks indicate significance at *F*-test *P<0.01 or **P<0.001 of the fitted regression line to the residuals over time (*n*=46 years of yield data, except for USA (*n*=47)). L, linear; QP, quadratic plateau; PW, piecewise with (+) increasing or (−) decreasing rate after breakpoint year; LUP/LLP, linear with upper/lower plateau; EXP, compound exponential; R, rainfed; I, irrigated.

Exponential models underperformed compared with linear models and exhibited large biases in the distribution of yield residuals (Figs 6, 7, 8 and Supplementary Table S7). Even in the few cases where exponential models performed well at describing yield trends, their residual error was comparable to that of the best-fit linear model (for example, maize and wheat yields in USA). Hence, there are three reasons why the use of exponential models to describe or project yield trajectories is not appropriate. First, where yields are increasing, absolute rates of yield increase are linear. Second, there is no case in which relative rates of yield gain have consistently increased or remained constant over time. Third, there are a number of cases where yield plateaus or a discontinuous break to slower rates of yield gain suggest the possibility of a biophysical ‘ceiling’ yield. In contrast, much of the published literature on yield projections is based on exponential rates of yield increase (Fig. 2 and Supplementary Table S2), which, as shown here, do not occur in the real world (Fig. 5). For example, a previous published study projected an average 1.4% per year compound rate of yield increase for irrigated rice in California from 2010 to 2050 (ref. 14) even when the average actual yield of California rice has not increased since 1990 (Fig. 5). Predicted irrigated rice yield in California by 2050, based on the above compound rate, is ~15,000 kg ha^−1^, which is >70% above the current average yield of ~9,000 kg ha^−1^.

## Discussion

Results from our analysis suggest that projections of crop yield trajectories based on extension of historical trends of the past five decades should be viewed with caution because these past trends were driven by rapid adoption of green revolution technologies that were largely one-time innovations. These include the development of semi-dwarf wheat and rice varieties, first widespread use of commercial fertilizers and pesticides, and large investments to expand irrigation infrastructure. A concern is that despite the increase in investment in agricultural R&D and education during this period^27^,^33^, the relative rate of yield gain for the major food crops has decreased over time together with evidence of upper yield plateaus in some of the most productive domains. For example, investment in R&D in agriculture in China has increased threefold from 1981 to 2000 (ref. 33). However, rates of increase in crop yields in China have remained constant in wheat, decreased by 64% in maize as a relative rate and are negligible in rice (Fig. 5 and Supplementary Table S5). Likewise, despite a 58% increase in investment in agricultural R&D in the United States from 1981 to 2000 (sum of public and private sectors), the rate of maize yield gain has remained strongly linear (Fig. 5 and Supplementary Table S5), implying that the marginal yield increase per unit of research investment has decreased substantially over time and highlighting the need for increasing the level of investment on agricultural R&D to sustain current and future increases in crop yields^33^.

These findings are consistent with the notion that as farmers’ yields move up towards the yield potential threshold, it becomes more difficult to sustain further yield gain because it requires fine tuning of many different facets of management in the production system^34^. Such fine tuning is often difficult to achieve in farmer’s fields, and the associated marginal costs, labour requirements, risks and environmental impacts may outweigh the benefits^34^. Therefore, substantial increases in future grain production will require significant increases in average crop yields in countries where current yield gaps are large as others have noted^35^. Paradoxically, many of the countries exhibiting the largest gaps have the poorest access to technology, infrastructure and capital required for agricultural development^36^.

To summarize, we found widespread deceleration in the relative rate of increase of average yields of the major cereal crops during the 1990–2010 period in countries with greatest production of these crops, and strong evidence of yield plateaus or an abrupt drop in rate of yield gain in 44% of the cases (Table 1), which, together, account for 31% of total global rice, wheat and maize production (Table 2). The results strongly support the proposition that estimates of future cereal production should be derived from yield projections based on linear models, with breakpoints and plateaus to reflect the linear nature of the crop yield gains in an ascending phase during which modern crop management practices are adopted, and the existence of a biophysical upper limit for grain yield best estimated by robust crop simulation models^3^. Approaches that rely on compound rates of yield increase or constant linear rates with no upper limit to yield growth are not supported by the analysis of historical yield trends and current understanding of crop physiology, and they are likely to overestimate future increases in crop yields by a large margin (Fig. 2 and Supplementary Table S2). In turn, overestimating trajectories in crop yields leads to estimates of land requirements for crop production that are too low and diminish capacity for effective strategic planning and research prioritization to ensure future food security and conservation of natural resources.

## Methods

### Overview

The time period of 1965–2010 over which yield trends were evaluated is the most relevant for use in estimating historical rates of yield gain to inform projections of future food security because it represents a contemporary period when public and private sectors in many developed and developing countries have invested heavily in application of modern genetics, agronomy and supporting basic sciences and information technologies to develop and gain adoption of technological advances in crop production.

Evaluated mathematical models were as follows: linear (L), quadratic with upper plateau (QP), linear piecewise (PW), linear with upper (LUP) or lower plateau (LLP), and compound-rate exponential (EXP) (Fig. 3). Except for the United States and Africa, where yield trends were analysed at the subnational and regional levels, respectively, the present study focuses on average yield at the national level because this is the spatial scale at which most projections of food security are made. Criteria for the selection of the best-fit trend are based on the assumption that the most appropriate statistical model is one that can describe the observed time trend in yield with the lowest error and minimal bias in distribution of residuals, and which uses the smallest possible number of estimated parameters while meeting the assumptions of normality, independence and homogenous variance in regression analysis^37^ (Fig. 4).

### Analysis of trends in cropland harvested area

Long-term (1965–2011) data in crop-harvested area were retrieved from FAOSTAT and used to analyse changes in cropland area of staple crops (cereal, oil, sugar, fibre, pulses, tuber and root crops) and three major cereal crops (rice, wheat and maize; FAOSTAT Database–Agricultural Production ( http://faostat.fao.org/). A three-phase linear model was fitted to the observed trends using the PROC NONLIN procedure in SAS Version 9.1 (SAS Institute, 9.1 Foundation for 64-bit Microsoft® Windows®, SAS Institute, Cary, NC):


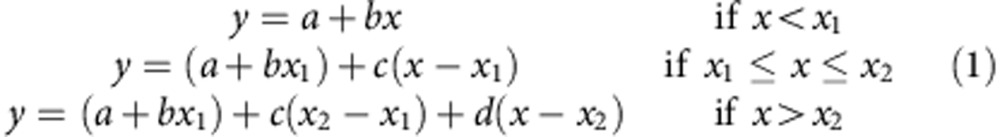


where *y* is crop-harvested area (Mha), *x* is year and *x*_1, 2_ are the breakpoint years. The fitted trilinear models have coefficient of determination (*r*^2^) of 0.97 and 0.93 (root mean square error (r.m.s.e.)=9.5 and 6.9 Mha) for area of staple crops and three major cereal crops, respectively. A total of 47 years of yield data were used in the regression analysis (1965–2011 time period). All estimated parameters were significant (Student’s *t*-test, *P*<0.01), except for parameter *c* in the fitted trend for cropland area of the three major cereal crops (Student’s *t*-test, *P*=0.20). An initial period of increase in harvested area occurred until 1980, followed by a period of little or no increase in harvested area of staple and major cereal crops, respectively, which lasted until early 2000s (Fig. 1). This period was followed by an unprecedented rate of expansion in harvested cropland area during the last decade, as indicated by the statistically significant higher rates of increase in harvested area of staple and major cereal crops during the last decade (parameter *d* in (equation 1)) compared with earlier rates of increase during the first two decades of the green revolution (parameters *b* and *c* in (equation 1); Student’s *t*-test; *P*<0.01).

Change in crop-harvested area during the last 10 years (2002–2011) was calculated for staple crops and, separately, for four crops (rice, wheat, maize and soybean) that accounted, altogether, for 83% of observed increase in staple crop area:


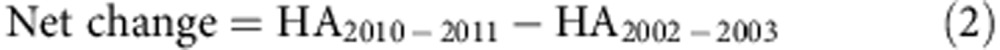



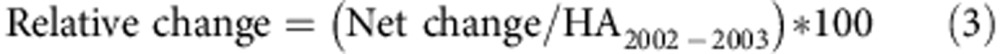


where net and relative changes are the absolute (Mha) and relative (%) differences in harvested area between two periods (Mha), and HA_2002–2003_ and HA_2010–2011_ are the calculated 2-year average harvested area for the 2002–2003 and 2010–2011 intervals, respectively. A net change of 85 Mha was observed for staple crops between 2002–2003 and 2010–2011 intervals, indicating that an 8% increase in global cropland area has occurred in only 10 years (Supplementary Table S1). This change was mostly accounted for by increased harvested area of maize and rice in Africa (+9 Mha), soybean in South America (+15 Mha) and maize, rice and soybean in South and Southeastern Asia (+16 Mha). In addition, remarkable was the little relative change of harvested crop areas (negative in some cases) observed for North and South Africa, West Asia, Europe and North America (Supplementary Table S1).

### Published projections in grain yields of cereal crop yields

Previously published projections on yield trajectories, based on exponential compound rates, were retrieved from the literature^12^,^13^,^14^,^15^,^16^,^17^,^18^ (Supplementary Table S2). In many of these projections, countries were grouped into categories according to their geographic location or degree of economic development, and a crop-specific exponential yield gain rate was assumed for each category. Other studies simplified this approach by assuming a single, worldwide exponential yield gain rate for each crop. A common feature of previous studies is the lack of recognition of a biophysical limit on crop yields determined by solar radiation, temperature and water supply from both rainfall and irrigation. To illustrate the discrepancy between reported projections and observed historical trends, reported projected trajectories for the US average maize yield were plotted and compared against the projected yields based on the historical (1965–2011) yield trend (Fig. 2).

### Data on grain yield data for cereal crops

Average annual yield data from 1965 to 2010 were retrieved for rice, wheat and maize in selected countries and regions, resulting in a total of 36 (crop–country or –region) cases that include a wide range of production environments and yield levels (FAOSTAT Database–Agricultural Production ( http://faostat.fao.org/; National Agricultural Statistics Service—Crops US state and county databases ( http://www.nass.usda.gov/index.asp; Supplementary Table S3). Available wheat and maize yield data in the USA also includes 2011 National Agricultural Statistics Service—Crops US state and county databases ( http://www.nass.usda.gov/index.asp). Hence, a total of 46 years of yield data were used for the yield trend analysis, except for the United States where a total of 47 years of yield data were used. In the case of the United States, trends were analysed for major producing regions: rice in California and south-central, wheat in the southern, central and northern Great Plains, and maize in the eastern and western Corn Belt. These regions accounted for 77, 47 and 85% of total US production of rice, wheat and maize, respectively. Separate analyses were performed for rainfed and irrigated maize yields in the western US Corn Belt, where both irrigated and rainfed production are important (54 and 46% of total maize production in western US Corn Belt, respectively). The analysis of maize yield trends in Africa was conducted for three regions: East, Central and West Africa. The average yield for a given region in the United States or Africa was calculated as the sum of total crop production in all the states (the United States) or countries (Africa) within the region divided by total harvested area.

### Statistical analysis of yield trends

Data on average annual grain yield were plotted against year for each crop–region case. Six statistical models, extensively used in the literature to describe yield trajectories, were evaluated for their performance of fitting trends in grain yields since the onset of the green revolution (Fig. 3):


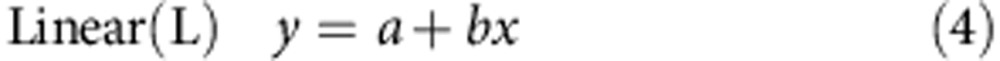



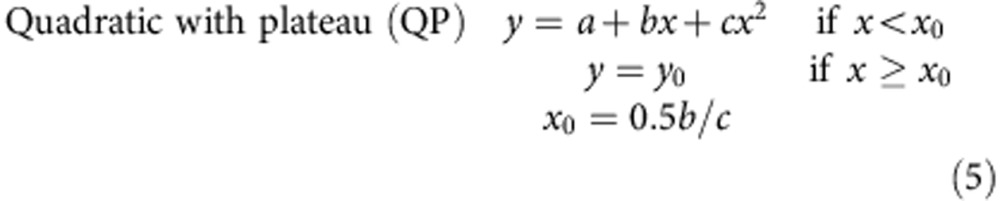



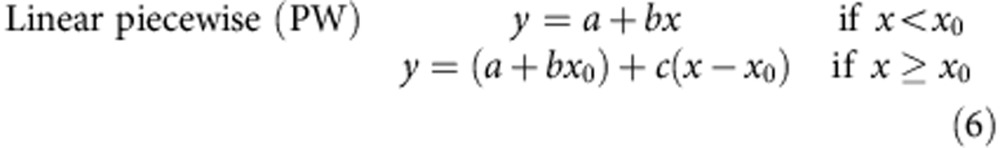



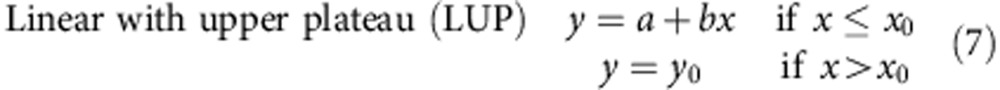



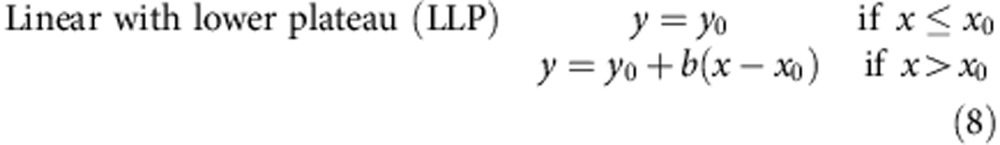



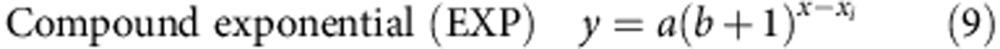


where *y* is grain yield (kg ha^−1^), *x* is year, *x*_i_ is the initial year of the time series (1965 in the present study) and *y*_0_ is the yield plateau level (kg ha^−1^).

SAS Version 9.1 programmes and procedures were used for all statistical analyses. The six models were fitted to observed yields using the PROC MIXED, PROC REG and PROC NONLIN procedures. Estimates of model coefficients (and associated confidence intervals), coefficient of determination (*r*^2^) and the r.m.s.e. were calculated for each crop–region model combination (Supplementary Tables S4 and S7). In the case of nonlinear models, *r*^2^ was calculated as the square correlation between predicted and observed values^38^. Violations of assumptions of regression analysis were identified by performing Shapiro–Wilks test for normality, Levene’s test for variance homogeneity and the Durbin–Watson’s (D) test for serial correlation^39^ (Supplementary Table S8). Residuals were plotted against year and the significance of linear- and quadratic models fitted to these plots was evaluated to detect potential biases along the time series (Figs 6, 7, 8). Finally, the Box–Cox method was used to identify suitable power transformations that can improve models performance^40^ (Supplementary Table S9).

The following criteria were used to identify the best-fit model for each crop–region yield trend: highly significant (Student’s *t*-test; *P*<0.01) model parameters, highest *r*^2^ and lowest r.m.s.e. compared with other models, and independent, normally distributed yield residuals with homogenous variance and unbiased distribution when residuals were plotted against year (Fig. 4). An additional criteria was that none of the applied data transformations improve model fit by >5% based on comparison of calculated *r*^2^ among models based on transformed and untransformed data^37^ (Fig. 4). In the process of selecting the best-fit model, the PW and LUP or LLP models were mutually exclusive, that is, the LUP or LLP models were discarded if estimated parameters of the PW model were all significant and no bias was detected in the distribution of yield residuals. In 22 out of the 36 cases, one statistical model clearly outperformed the others by a difference in r.m.s.e. ≥5% and this was the chosen best-fit model (Supplementary Tables S4 and S7). When differences in r.m.s.e. among two or more models were <5% (14 out of 36 cases), the two models with lowest r.m.s.e. were selected as best-fit models, unless there was a justification to discard one of them, for example, failure to meet assumptions of regression analysis (EXP in Canada) or when the model with higher r.m.s.e. also had the larger number of parameters (QP for wheat in the Netherlands and maize in Italy).

Selected best-fit models exhibited highly significant coefficients (Student’s *t*-test; *P*<0.01) and higher goodness of fit compared with other fitted models (higher *r*^2^ and smaller r.m.s.e.; Supplementary Tables S4 and S7). Out of 45 selected best-fit models, only 1 and 4 did not meet assumptions of variance homogeneity (LLP model for rice in Vietnam) and normality (L and EXP models for maize in the United States), respectively (Levene and Shapiro–Wilks tests; *P*<0.01) (Supplementary Table S8). In contrast, ten selected best-fit models exhibited significant positive serial autocorrelation: rice in Bangladesh, China, Indonesia, Republic of Korea, Philippines, Vietnam, wheat in China and maize in central and west Africa and Italy (D-test; *P*<0.01; Supplementary Table S8). Residual plots of the best-fit models did not exhibit any obvious trends over time, except for four cases with severe positive serial autocorrelation: rice and wheat in China and rice in Indonesia and Philippines (0.45<D<0.80), which are possibly related to vigorous public sector varietal improvement and agronomic research programmes that promote rapid adoption of improved rice varieties and associated fertilizer and pest management practices and/or the frequency of the yield survey (Figs 6, 7, 8 and Supplementary Table S8). Serial correlation affects the estimated variances but does not affect the value of the estimated model coefficients, which results in the estimators looking more accurate than they actually are^38^. To test how serial correlation may have affected the significance of the estimators, we assumed the estimated variance to be 60% of the true variance, which is expected for a first-order serial correlation coefficient (AR(1)) of 0.5 (ref. 39), similar to the estimated AR(1) of 0.6 found for the above four crop–country cases. Results indicate that the parameter estimates of the best-fit models were still highly significant despite serial correlation (Student’s *t*-test; *P*<0.01). EXP models exhibited a remarkable bias on their residuals plots in 75% of the 36 cases (with significant positive autocorrelation in 58% of the cases) and did not meet assumptions of regression analysis in 31% of the cases (Figs 6, 7, 8 and Supplementary Table S8).

Other evidence of the robustness of selected best-fit models was that their fit, based on the original untransformed data, was not improved after data transformation using the Box–Cox method^40^, except for rice in Republic of Korea and maize in east Africa (Supplementary Table S9). In both cases, model fitness increased slightly after applying a reciprocal transformation to the yield data (*r*^2^=0.90 versus 0.84 (Republic of Korea) and 0.57 versus 0.49 (East Africa), with and without data transformation, respectively). None of the non-selected models fit to the transformed data outperformed the fit of the selected models shown in Table 1. And although the presence of abnormally low- or high-yield years during the last years of the time series can potentially hinter the identification of yield plateaus, this is not a large concern in the present study because all cases that exhibited an upper yield plateau have ≥10 years of yield data after the breakpoint year, except for maize in France with 7 years. Perhaps, more important, the 99% confidence interval of the breakpoint year (*x*_0_) was within the 1965–2010 interval in all cases. In addition, visual inspection of the crop–country trends with statistically significant yield plateaus did not have unusually high or low yields around the breakpoint year. Therefore, it is unlikely that the upper yield plateaus identified in the present study are the consequence of a few yield outliers at the end of the time series. The presence of autocorrelation and changes in variance over time can also hinder the identification of yield plateaus^29^. However, out of the 21 selected LUP or LLP models in the present study, variance was not homogenous in only one case (rice in Vietnam) and autocorrelation was significant in six cases (rice in China, Republic of Korea and Vietnam, and maize in central and west Africa, and Italy) (Supplementary Table S8). And even in the six cases with significant autocorrelation or non-homogenous variance, visual inspection of the residuals clearly indicated that LUP or LLP models outperform linear models (Figs 6, 7, 8). Although the yield residual versus time relationship of the linear model exhibited a strong trend, there was no detectable pattern in the yield residuals of the LUP or LLP models, with the only exception of maize in West Africa where there was no detectable trend in both linear and LLP models.

Yield trends from cases in which there was evidence of yield plateaus were re-analysed to determine the number of years after the breakpoint year (*x*_0_) that were needed to identify a yield plateau (hereafter called *x*_n_). The LUP model was successively fitted to yield trends in which data-years after *x*_0_ were removed and then added one by one until estimated *x*_0_ became statistically significant (Student’s *t*-test; *P*<0.01). Values of *x*_n_ varied across cases, depending on the magnitude of year-to-year variation in yields along the entire trend line (Supplementary Table S6). In fact, there was a negative relationship between *x*_n_ and *r*^2^ of the fitted LUP model to each crop–country case (*y*=20−14.9 *x*; *r*^2^=0.63; Student’s *t*-test; *P*<0.01). A conservative value of *x*_n_=7 years can be taken as the minimum number of years needed, after the breakpoint year, to detect a statistically significant yield plateau in production systems that exhibited high *r*^2^ (>0.85) such as rice in China and California, wheat in northwestern Europe and maize in Italy and France. In production systems with larger variability in yield along the yield trends, a greater number of years is required, ranging from 9 years in Japan (*r*^2^=0.63) to 17 years in East Africa (*r*^2^=0.49). The total number of years with 'flat' yields required to detect a statistically significant yield plateau, including the breakpoint year, was calculated as *x_n_*+1 and this is the value reported in the Results section.

### Calculation of absolute and relative yield gain rates

Absolute yield gain rates (kg ha^−1^ per year) were calculated from the first derivative of the best-fit model, for each crop–region case, at three points in the time interval: 1970, 1990 and 2010 (Supplementary Table S5). For those cases in which there were two best-fit models, the model with lowest r.m.s.e. was chosen to describe the yield trend. Despite the slightly better performance of the EXP model, the L model was chosen to describe trends in maize yield in the United States because fit of the EXP and L models was very similar (difference in r.m.s.e. ≤2%), there was no evidence of changes in the absolute yield gain rate between 1965 to 2011 as tested using piecewise with one, two or three breakpoint years and the EXP model underperformed compared with other models in all the other cases. Moreover, using an exponential model would imply predicted average maize yields by 2030 that are 35 and 37% higher than the 2010 yield for maize in the eastern and (irrigated) western US Corn Belt, which will require average (2010–2030) yield gain rates of 180 and 220 kg ha^−1^ per year. These rates are 50 and 70% higher than the observed yield gain rates during the 1965–2010 interval. This is unlikely to occur given the slow down in yield gain rates observed in other intensive cropping systems and lack of increase observed for irrigated maize in contest winners and farmers’ fields in the western US Corn Belt^3^,^19^. In fact, although no yield plateau was detected for irrigated maize in the western US Corn Belt for the 1965–2011 interval, the linear regression for the last 10-year period (2002–2011) has a slope indistinguishable from zero (Student’s *t*-test; *P*=0.26, *n*=10).

Relative rates of yield gain (% per year) were calculated for 1970, 1990 and 2010 as the ratio between the absolute yield gain rate and the yield-trend-predicted yield in the particular year, expressed as percentage (Supplementary Table S5). Exponential compound rates (% per year) were also derived for each crop–region case from the parameter *b* in (equation 9), expressed as percentage. The objective was to highlight the limitations of using compound rates of yield gain to predict future yield trajectories: out of a total of 29 cases that have compound rate >1% per year, there was evidence of yield plateaus or decreasing yield gain rates in 41% of the cases, decreasing relative rates of yield gain during the entire time series in 66% of the cases and decrease in the relative rate of yield gain in the time interval between 1990 and 2010 in all cases.

## Author contributions

P.G. and K.G.C. conceived the study and wrote the paper. P.G. and K.M.E. performed the statistical analysis. All authors contributed to editing the paper.

## Additional information

**How to cite this article:** Grassini, P. *et al.* Distinguishing between yield advances and yield plateaus in historical crop production trends. *Nat. Commun.* 4:2918 doi: 10.1038/ncomms3918 (2013).

## Supplementary Material

Supplementary Information Supplementary Tables S1-S9

## Figures and Tables

**Table 1:** Best-fit models identified for each crop and country or region.

**Country/region**	**Crops**		
	**Rice**	**Wheat**	**Maize**
*Africa*			
East			LUP*
Central			LLP
West			LLP
			
*America*			
Argentina		L, EXP	PW+, EXP
Brazil			PW+
Canada		LLP	
United States			
California	LUP		
South-central	LLP		
South Great Plains		LUP	
Central Great Plains		L, EXP	
North Great Plains		LLP, EXP	
Eastern corn belt			L, EXP
Western Corn Belt			
Irrigated			L, EXP
Rainfed			L, EXP
			
*Asia*			
Bangladesh	PW+		
China	LUP	L	PW−
India	L	LUP	LLP
Indonesia	PW−		
Japan	L, LUP		
Korea	LUP *		
Philippines	L		
Thailand	PW+		
Vietnam	LLP		
			
*Europe*			
Denmark		LUP	
France		LUP	LUP
Germany		LUP	
Italy			LUP
The Netherlands		LUP	
United Kingdom		LUP	
			
*Oceania*			
Australia		L, EXP	

L, linear (no plateau); QP, quadratic plateau; PW, piecewise with (+) increasing or (−) decreasing rate after breakpoint year; LUP or LLP, linear with upper or lower plateau; EXP, compound exponential.

^*^Box–Cox procedure indicates that reciprocal transformation improves model fit.

**Table 2:** Percentage of global crop production under different crop yield trajectories.

**Crop species**	% of global production*	**Increasing rate (%)**†	**Constant rate (%)**	**Decreasing rate (%)**	**Upper yield plateaus (%)**
Rice	84	19	23	9	33
Wheat	56	5	24	0	27
Maize	71	13	33	20	5
Total rice, wheat and maize	70	12	27	10	21

^*^Percentage of global production of rice, wheat and maize, and the three crops together accounted for by the 36 crop–country cases analysed in this study.

^†^The 36 crop–country cases were grouped according to the pattern of change in rates of crop yield gains during the 1965–2010 interval and total production was calculated for each group and for each crop.

## References

[b1] Royal Society of London. Reaping the Benefits: Science and the Sustainable Intensification of Global Agriculture Royal Society: London, (2009).

[b2] FAO. The State of The World’s Land and Water Resources for Food and Agriculture (SOLAW)–Managing Systems at Risk FAO: Rome and Earthscan, London, (2011).

[b3] Van Ittersum M. K. *et al.* Yield gap analysis with local to global relevance—a review. Field Crops Res. 143, 4–17 (2013).

[b4] Burney J., Davis S. J. & Lobell D. B. Greenhouse gas mitigation by agricultural intensification. Proc. Natl Acad. Sci. USA 107, 12052–12057 (2010). 20551223 10.1073/pnas.0914216107 PMC2900707

[b5] Vermeulen S. J., Campbell B. M. & Ingram J. S. I. Climate change and food systems. Annu. Rev. Environ. Resource 37, 195–222 (2012).

[b6] Fischer R. A. & Edmeades G. O. Breeding and cereal yield progress. Crop Sci. 50, 85–98 (2010).

[b7] Hafner S. Trends in maize, rice, and wheat yields for 188 nations over the past 40 years: a prevalence of linear growth. Agri. Ecosyst. Environ. 97, 275–283 (2003).

[b8] Searchinger T. R. *et al.* Use of U.S. croplands for biofuels increases greenhouses gases through emissions from land-use change. Science 319, 1238–1240 (2008). 18258860 10.1126/science.1151861

[b9] Jaggard K. W., Qi A. & Ober E. S. Possible changes to arable crop yields by 2050. Phil. Trans. R Soc. B 365, 2835–2851 (2010). 20713388 10.1098/rstb.2010.0153 PMC2935124

[b10] Dyson T. World food trends and prospects to 2025. Proc Natl Acad Sci U.S.A. 96, 5929–5936 (1999). 10339520 10.1073/pnas.96.11.5929 PMC34208

[b11] Ray D. K., Mueller N. D., West P. C. & Foley J. A. Yield trends are insufficient to double global crop production by 2050. PLoS ONE 8, e66428 (2013). 23840465 10.1371/journal.pone.0066428 PMC3686737

[b12] Evenson R. & Rosegrant M. W. Productivity Projections for Commodity Market Modeling Yale University Economic Growth Center: New Haven, (1995).

[b13] Evenson R. E. Global and local implications of biotechnology and climate change for future food supplies. Proc. Natl Acad. Sci. USA 96, 5921–5928 (1999). 10339519 10.1073/pnas.96.11.5921 PMC34207

[b14] Nelson G. C. *et al.* Food Security, Farming, and Climate Change to 2050 IFPRI: Washington, DC, (2010).

[b15] Reilly J. M. & Fuglie K. O. Future yield growth in field crops: what evidence exists? Soil Till. Res. 47, 275–290 (1998).

[b16] Heisey P. W. Amber Waves USDA-ERS Publication (2009) http://webarchives.cdlib.org/sw1vh5dg3r/http://ers.usda.gov/AmberWaves/December09/Features/USCornYields.htm.

[b17] Edgerton M. D. Increasing crop productivity to meet global needs for feed, food, and fiber. Plant Phys. 149, 7–13 (2009). 10.1104/pp.108.130195 PMC2613695 19126690

[b18] Hertell T. W. *et al.* Global land use and greenhouse gas emissions impacts of maize ethanol: the role of market-mediated responses. Biosci. 60, 223–231 (2010).

[b19] Duvick D. N. & Cassman K. G. Post-green revolution trends in yield potential of temperate maize in the North-Central United Sates. Crop Sci. 39, 1622–1630 (1999).

[b20] Specht J. E., Hume D. J. & Kumudini S. V. Soybean yield potential - A genetic and physiological perspective. Crop Sci. 39, 1560–1570 (1999).

[b21] Peng S., Cassman K. G., Virmani S. S., Sheehy J. & Khush G. S. Yield potential trends of tropical rice since the release of IR8 and the challenge of increasing rice yield potential. Crop Sci. 39, 1552–1559 (1999).

[b22] Graybosch R. A. & Peterson C. J. Genetic improvement in winter wheat yields in the Great Plains of North America, 1959–2008. Crop Sci. 50, 1882–1890 (2010).

[b23] Lobell D. B., Cassman K. G. & Field C. B. Crop yield gaps: their importance, magnitude, and causes. Annu. Rev. Environ. Resource 34, 1–26 (2009).

[b24] Evans L. T. Crop Evolution, Adaptation, and Yield Cambridge University Press: Cambridge, (1993).

[b25] Bruinsma J. The Resource Outlook to 2050: By How Much do Land, Water Use and Crop Yields Need to Increase by 2050? ed Conforti P. chapter 6FAO: Rome, (2011).

[b26] Alexandratos N. & Bruinsma J. World Agriculture Towards 2030/2050: the 2012 Revision. ESA Working Paper No. 12-03 FAO: Rome, (2012).

[b27] Alston J. M., Beddow J. M. & Pardey P. G. Agricultural research, productivity, and food prices in the long run. Science 325, 1209–1210 (2009). 19729642 10.1126/science.1170451

[b28] Ray D. K. *et al.* Recent patterns of crop yield growth and stagnation. Nat. Commun. 3, 1293 (2012). 23250423 10.1038/ncomms2296

[b29] Lin M. & Huybers P. Reckoning wheat yield trends. Environ. Res. Lett. 7, 024016 (2012).

[b30] Anderson J. R. & Hazell P. B. R. Implications for Agricultural Research and Policy in Developing Countries The Johns Hopkins University Press: Baltimore and London, (1989).

[b31] Brisson N. *et al.* Why are wheat yields stagnating in Europe? A comprehensive data analysis for France. Field Crops Res. 119, 201–212 (2010).

[b32] Rijk B., van Ittersum M. & Withagen J. Genetic progress in Dutch crop yields. Field Crop Res. 149, 201.

[b33] Pardey P. G., Beintema N., Dehmer S. & Wood S. Agricultural Research. A Growing Global Divide? International Food Policy Research Institute: Washington, DC, (2006).

[b34] Cassman K. G., Dobermann A., Walters D. T. & Yang H. S. Meeting cereal demand while protecting natural resources and improving environmental quality. Annu. Rev. Environ. Resource 28, 315–358 (2003).

[b35] Foley J. A. *et al.* Solutions for a cultivated planet. Nature 478, 337–342 (2011). 21993620 10.1038/nature10452

[b36] Pardey P. G., Alston J. M. & Piggott R. R. Agricultural R&D in the Developing World: Too Little, Too Late? International Food Policy Research Institute: Washington, DC, (2006).

[b37] Archontoulis S. V. & Miguez F. A. Nonlinear regression models and application in agricultural research. Agron. J. 105, 1–13 (2013).

[b38] Gallant A. R. Nonlinear Statistical Models John Willey: New York, (1989).

[b39] Johnston J. & Di Nardo J. Econometric Methods McGraw Hill: New York, (1997).

[b40] Box G. E. P. & Cox D. R. An analysis of transformations. J. R. Stat. Soc. B Met. 26, 211–252 (1964).

