# Supplementary material for: Distinguishing between yield advances and yield plateaus in historical crop production trends
Source: Nat Commun. 2013 Dec 17;4:2918. doi: 10.1038/ncomms3918 (PMC3905725; doi:10.1038/ncomms3918)
Supplement: Supplementary Information — Supplementary Tables S1-S9 [file ncomms3918-s1.pdf]

Supplementary information

Supplementary Table S1 Change in total harvested area of staple crops and selected grain crops by continent and sub-regions as defined by the Food and Agriculture Organization.

	Staple crops *	Rice (R)	Wheat (W)	Maize (M)	Soybean (S)	Total R+W+M+S
World	+84.6 (+8%)	+14.9 (+7%)	+8.1 (+5%)	+26.2 (+17%)	+21.5 (+25%)	+70.7 (+12%)
Africa †	+16.9 (+10%)	+2.9 (+37%)	+0.4 (+5%)	+6.4 (+24%)	+0.3 (+26%)	+10.0 (+22%)
East	*+11.0 (+25%)*	*+1.4 (+63%)*	*+0.7 (+47%)*	*+2.8 (+24%)*	*+0.1 (+24%)*	*+4.9 (+31%)*
Middle	*+4.2 (+29%)*	*+0.1 (+25%)*	*nil*	*+1.4 (+46%)*	*nil*	*+1.5 (+42%)*
North	*-5.3 (-19%)*	*-0.1 (-18%)*	*-4.4 (-22%)*	*nil*	*nil*	*-0.1 (-1%)*
South	*-1.2 (-17%)*	*nil*	*-0.3 (-32%)*	*-1.0 (-26%)*	*+0.3 (+225%)*	*-1.0 (-21%)*
West	*+8.2 (+11%)*	*+1.6 (+34%)*	*nil*	*+3.2 (+47%)*	*-0.1 (-13%)*	*+4.7 (+39%)*
America ‡	+22.4 (+10%)	*nil*	-3.4 (-9%)	+7.1 (+13%)	+16.8 (+27%)	+20.5 (+12%) [c]
North	*+1.6 (+1%)*	*nil*	*-2.2 (-7%)*	*+5.0 (+17%)*	*+1.5 (+5%)*	*+4.4 (+5%)*
Central and Caribbean	*-0.8 (-4%)*	*nil*	*+0.1 (+8%)*	*-0.2 (-2%)*	*+0.1 (+114%)*	*nil*
South (total)	*+21.5 (+25%)*	*nil*	*-1.2 (-13%)*	*+2.3 (+13%)*	*+15.1 (+48%)*	*+16.2 (+25%)*
Tropical and subtropical §	*+13.5 (+22%)*	*-0.1 (-3%)*	*+0.1 (+4%)*	*+1.3 (+8%)*	*+7.8 (+40%)*	*+9.0 (+21%)*
Temperate ¶	*+8.0 (+31%)*	*+0.1 (+40%)*	*-1.4 (-21%)*	*+1.0 (+40%)*	*+7.3 (+61%)*	*+7.1 (+33%)*
Asia †	+41.9 (+9%)	+11.8 (+9%)	+6.1 (+6%)	+11.6 (+27%)	+2.7 (+16%)	+32.2 (+11%)
Central	*+2.4 (+12%)*	*nil*	*+2.0 (+14%)*	*nil*	*nil*	*+1.9 (+13%)*
East ‡	*+7.8 (+6%)*	*+2.3 (+8%)*	*+1.4 (+6%)*	*+8.7 (+35%)*	*-1.0 (-10%)*	*+11.4 (+13%)* [c]
South	*+19.3 (+9%)*	*+2.9 (+5%)*	*+4.7 (+11%)*	*+1.3 (+14%)*	*+3.5 (+54%)*	*+12.3 (+11%)*
Southeast	*+15.5 (+19%)*	*+6.6 (+15%)*	*nil*	*+1.6 (+20%)*	*+0.2 (+16%)*	*+8.4 (+16%)*
West	*-3.1 (-11%)*	*nil*	*-1.9 (-13%)*	*nil*	*Nil*	*-1.9 (-12%)*
Europe	+1.9 (+1%)	+0.2 (+27%)	+3.5 (+7%)	+1.2 (+8%)	+1.8 (+160%)	+6.6 (+9%)
East ‡	*+4.4 (+4%)*	*+0.1 (+58%)*	*+3.4 (+10%)*	*+1.8 (+23%)*	*+1.7 (+249%)*	*+7.0 (+16%)*
North	*+0.1 (+1%)*	*nil*	*+0.6 (+17%)*	*nil*	*Nil*	*+0.6 (+17%)*
South	*-3.1 (-12%)*	*+0.1 (+14%)*	*-1.7 (-24%)*	*-0.5 (-13%)*	*+0.1 (+24%)*	*-2.0 (-18%)*
West	*+0.5 (+2%)*	*nil*	*+1.2 (+13%)*	*-0.2 (-7%)*	*Nil*	*+1.0 (+9%)*
Oceania	+1.4 (+6%)	-0.1 (-49%)	+1.4 (+12%)	*nil*	*Nil*	+1.3 (+11%)

.

Note that absolute change (in Mha) was calculated as the difference between the 2-y crop harvested area averages calculated for the 2010-2011 and 2002-2003 intervals. Percentage change is shown in parenthesis. * Includes all cereal, oil, sugar, pulses, fiber, tuber, and root crops. † Remaining increase in total crop land area in Africa and Asia, not explained by R+W+M+S, is mostly accounted by groundnuts (+1.7 Mha), dry beans (+1.3 Mha), cassava (+1.2 Mha), and cowpea (+1.0 Mha) in Africa and oil palm (+4.0 Mha), seed cotton (+3.8 Mha), and chickpea (+2.3 Mha) in Asia. ‡ Greater increase of R+W+M+S cropland area than in total cropland area in America, East Asia, and East Europe is explained by expansion of R+W+M+S at expense of harvested area of sorghum (-1.3 Mha), cotton (-0.8 Mha), and barley (-0.8 Mha) in USA, sweet potatoes (-1.7 Mha), dry beans (-0.4 Mha), and groundnuts (-0.4 Mha) in East Asia, and barley (-3.7 Mha), rye (-2.6 Mha), and potatoes (-1.8 Mha) in East Europe, respectively. § Brazil, Colombia, Peru, Guyana, Suriname, French Guiana, Ecuador, Venezuela, Bolivia, and Paraguay. ¶ Argentina, Uruguay, and Chile.

Supplementary Table S2 Examples of compound annual rates of yield increase used in previous studies that evaluated scenarios of future food production security.

Reference	Country	Crop species	Water regime	Reported projected annual yield gain rate (% per year)*	Projected time interval
(12)	South Asia	Rice	Total †	2.3	1995-2020
		Wheat/Maize		1.9	
(13)	United States	Cereals	Total †	1.0	1993-2020
	Western Europe			0.4	
	Japan			-0.03	
	Australia			1.8	
	Latin America			1.4	
	Central-west Africa			1.8	
	Eastern Africa			1.8	
	Sub-Saharan Africa			1.7	
	India			1.4	
	Bangladesh			1.4	
	Indonesia			1.0	
	Thailand			1.0	
	Philippines			1.0	
	Vietnam			1.4	
	China			1.0	
(14)	California	Rice	Rainfed	0.8	2010-2050
			Irrigated	1.4	
	Developed countries	Rice	Rainfed	0.7	
			Irrigated	0.8	
		Wheat	Rainfed	1.1	
			Irrigated	1.4	
		Maize	Rainfed	0.7 (2.0) ‡	
			Irrigated	0.4 (2.0) ‡	
	Low-income countries	Rice	Rainfed	0.6	
			Irrigated	0.6	
		Wheat	Rainfed	1.9	
			Irrigated	1.5	
		Maize	Rainfed	1.0	
			Irrigated	0.4	
	Mid-income countries	Rice	Rainfed	0.8	
			Irrigated	0.6	
		Wheat	Rainfed	1.3 (2.0) ‡	
			Irrigated	0.7 (2.0) ‡	
		Maize	Rainfed	1.0 (2.0) ‡	
			Irrigated	0.9 (2.0) ‡	
(15)	United States	Rice	Total †	2.1	1995-2020
		Wheat		1.8	
		Maize		2.9	
(16)	United States	Maize	Total †	2.9	2001-2030
(17)	United States	Maize	Total †	3.6	2010-2030
(18)	United States	Maize	Current cropland	2.8	2001-2015

* Compound growth rates calculated from values for the begin- and end-point of the projected time interval. † Not disaggregated by rainfed and irrigated regimes. ‡ Reported yield gain rate (% per year) under a scenario of higher investment on agriculture R&D is shown in parenthesis.

Supplementary Table S3 Countries and regions selected for analysis of yield trends for the three major cereal crops from 1965 to 2010.

Rice (669 million Mg)	Wheat (647 million Mg)	Maize (798 million Mg)
Bangladesh (7%)	Argentina (2%)	Africa:
China (29%)	Australia (3%)	east (2%)
India (21%)	Canada (4%)	central (<1%)
Indonesia (9%)	China (17%)	west (2%)
Japan (2%)	Denmark (1%)	Argentina (2%)
Korea, Rep. of (2%)	France (6%)	Brazil (7%)
Philippines (2%)	Germany (4%)	China (20%)
Thailand (5%)	India (12%)	France (2%)
United States:	The Netherlands (<1%)	India (2%)
California, (<1%)	United Kingdom (2%)	Italy (1%)
south-central (1%)	US Great Plains:	US Corn Belt:
Vietnam (6%)	south (1%)	east, rainfed (24%)
	central (2%)	west, irrigated (5%)
	north (2%)	west, rainfed (4%)
Cumulative portion of annual global production:		
Rice = 84%	Wheat = 56%	Maize = 71%

Note that average (2006-2010) world total production and percentage accounted by each country are shown in parenthesis for each crop. USA. South-central: Missouri, Mississippi, Arkansas, Louisiana, and Texas; US Great Plains: Oklahoma and Texas (south), Nebraska and Kansas (central); South Dakota and North Dakota (north); US Corn Belt: Iowa, Illinois, Minnesota, Indiana, Ohio, and Missouri (east); Nebraska, Kansas, South Dakota, North Dakota, Wyoming, Colorado, Texas, and Oklahoma (west). East Africa: Burundi, Djibouti, Eritrea, Ethiopia, Kenya, Madagascar, Malawi, Mozambique, Rwanda, Somalia, Uganda, Tanzania, Zambia, and Zimbabwe; Central Africa: Angola, Cameroon, Central Africa, Chad, Congo, Democratic Congo, Equatorial Guinea, Sao Tome and Principe, and Gabon; West Africa: Benin, Burkina Faso, Cape Verde, Cote d'Ivoire, Gambia, Ghana, Guinea, Guinea Bissau, Liberia, Mali, Mauritania, Niger, Nigeria, Senegal, Sierra Leone, and Togo.

Supplementary Table S4 Selected statistical models for each crop-region case and associated goodness of fit and coefficients.

Country/region	Model	r^2 (*r.m.s.e.*) *	Estimated model parameters †				
			a (kg ha^{-1})	b (kg ha^{-1} per year)	c (kg ha^{-1} per year)	x_0	y_0 (kg ha^{-1})
Rice							
Bangladesh	PW+	0.98 (120)	-55,143	28.9	88.7	1988	
China	LUP	0.97 (216)	-221,441	114.1		1997	6,394
India	L	0.95 (142)	-87,503	45.2			
Indonesia	PW-	0.98 (130)	-214,770	110.2	31.1	1987	
Japan	L	0.61 (294)	-47,214	26.8			
	LUP	0.63 (290)	-61,543	34.1		1997	6,466
Korea, Rep. of	LUP	0.81 (412)	-409,101	210.0		1978	6,363
Philippines	L	0.95 (167)	-106,355	54.8			
Thailand	PW+	0.95 (89)	-18,213	10.2	45.2	1988	
United States:							
California	LUP	0.87 (490)	-262,791	136.4		1992	8,822
south-central	LLP	0.93 (266)		95.8		1980	4,787
Vietnam	LLP	0.99 (134)		106.0		1979	2,013
Wheat							
Argentina	L	0.80 (232)	-66,766	34.6			
	EXP	0.80 (234)	1,277	0.018			
Australia	L	0.23 (336)	-24,754	13.2			
	EXP	0.22 (333)	1,202	0.009			
Canada	LLP	0.63 (248)	1,734	30.7		1980	
China	L	0.97 (198)	-173,723	88.8			
Denmark	LUP	0.84 (477)	-198,477	103.1		1995	7,208
France	LUP	0.93 (387)	-242,219	124.9		1996	7,081
Germany	LUP	0.94 (365)	-229,670	118.6		2000	7,494
India	LUP	0.98 (94)	-103,404	53.1		2000	2,727
The Netherlands	LUP	0.90 (503)	-302,080	155.8		1993	8,445
United Kingdom	LUP	0.91 (451)	-265,606	137.0		1996	7,805
US Great Plains:							
south	LUP	0.30 (280)	-66,136	34.4		1983	2,005
central	L	0.37 (346)	-35,417	19.0			
	EXP	0.36 (349)	1,948	0.008			
north	LLP	0.50 (312)		27.9		1980	1,705
	EXP	0.48 (314)	1,546	0.011			
Maize							
Africa:							
east	LUP	0.49 (128)	-37,230	19.5		1984	1,390
central	LLP	0.73 (56)		13.0		1989	761
west	LLP	0.91 (96)		28.4		1979	855
Argentina	PW+	0.91 (512)	-134,778	69.6	175.8	1989	
	EXP	0.90 (526)	1,901	0.030			
Brazil	PW+	0.96 (183)	-52,845	27.5	104.9	1990	
China	PW-	0.97 (211)	-225,006	115.2	41.5	1993	
France	LUP	0.91 (536)	-266,056	137.3		2003	8,901
India	LLP	0.90 (134)		35.5		1978	1,045
Italy	LUP	0.96 (380)	-328,265	169.0		1997	9,347
US Corn Belt:							
east, rainfed	L	0.82 (774)	-227,037	118.0			
	EXP	0.83 (757)	5,213	0.016			
west, irrigated	L	0.93 (491)	-246,713	128.6			
	EXP	0.93 (490)	6,280	0.015			
west, rainfed	L	0.64 (879)	-161,676	83.9			
	EXP	0.65 (867)	3,359	0.017			

L: linear; QP: quadratic plateau; PW: piecewise with (+) increasing or (-) decreasing rate after breakpoint year; LUP/LLP: linear with upper/lower plateau; EXP: compound exponential. Forty-six years of yield data were used in the regression analyses (1965-2010) except for USA (n = 47, 1965-2011).

* Coefficient of determination (r^2) and root mean square error (r.m.s.e., in kg ha^{-1}).

† Estimated model parameters were all significant (Student's t-test; P<0.01). See model equations in the Methods section.

Supplementary Table S5 Linear rates of increase in yield. Rates of increase in yield are relative to the trend-line yield in 1970, 1990, and 2010.

Country/region	Trend-line yields (kg ha^{-1}) *			Absolute (kg ha^{-1} per year) and relative rate of gain (% per year, in parenthesis) †			Compound rate (% per year) ‡
	1970	1990	2010	1970	1990	2010	
Rice							
Bangladesh (PW+)	1,790	2,488	4,262	29 (1.6)	89 (3.6)	89 (2.1)	2.5
China (LUP)	3,336	5,618	6,394	114 (3.4)	114 (2.0)	0 (0)	1.7
India (L)	1,541	2,445	3,349	45 (2.9)	45 (1.8)	45 (1.3)	1.9
Indonesia (PW-)	2,324	4,291	4,913	110 (4.7)	31 (0.7)	31 (0.6)	1.8
Japan (LUP)	5,434	6,344	6,722	34 (0.6)	34 (0.5)	0 (0)	0.4
Korea, Rep. (LUP)	4,599	6,363	6,363	210 (4.6)	0 (0)	0 (0)	0.9
Thailand (PW+)	1,881	2,155	3,059	10 (0.5)	45 (2.1)	45 (1.5)	1.3
Philippines (L)	1,601	2,697	3,793	55 (3.4)	55 (2.0)	55 (1.5)	2.1
United States:							
California (LUP)	5,917	8,645	8,822	136 (2.3)	136 (1.6)	0 (0)	1.1
south-central (LLP)	4,787	5,745	7,661	0 (0)	96 (1.7)	96 (1.3)	1.3
Vietnam (LLP)	2,013	3,179	5,299	0 (0)	106 (3.3)	106 (2.0)	2.9
Wheat							
Argentina (L)	1,396	2,088	2,780	35 (2.5)	35 (1.7)	35 (1.3)	1.8
Australia (L)	1,250	1,514	1,778	13 (1.9)	13 (0.9)	13 (0.7)	0.9
Canada (LLP)	1,734	2,041	2,655	0 (0)	31 (1.5)	31 (1.2)	1.2
China (L)	1,213	2,989	4,765	89 (7.3)	89 (3.0)	89 (1.9)	3.0
Denmark (LUP)	4,630	6,692	7,208	103 (2.2)	103 (1.5)	0 (0)	1.2
France (LUP)	3,834	6,332	7,081	125 (2.7)	125 (1.9)	0 (0)	1.6
Germany (LUP)	3,972	6,344	7,494	119 (3.0)	119 (1.9)	0 (0)	1.7
India (LUP)	1,203	2,265	2,727	53 (4.4)	53 (2.3)	0 (0)	2.3
The Netherlands (LUP)	4,846	7,962	8,445	156 (3.2)	156 (2.0)	0 (0)	1.5
United Kingdom (LUP)	4,284	7,024	7,805	137 (3.2)	137 (2.0)	0 (0)	1.6
US Great Plains:							
south (LUP)	1,632	2,005	2,005	34 (2.1)	0 (0)	0 (0)	0.6
central (L)	2,013	2,393	2,773	19 (0.9)	19 (0.8)	19 (0.7)	0.8
north (LLP)	1,705	2,292	2,850	0 (0)	28 (1.2)	28 (1.0)	1.1
Maize							
Africa:							
eastern (LUP)	1,185	1,390	1,390	20 (1.6)	0 (0)	0 (0)	0.6
central (LLP)	761	774	1,034	0 (0)	13 (1.7)	13 (1.3)	0.7
west (LLP)				0 (0)	28 (2.4)	28 (1.6)	2.0
Argentina (PW+)	2,334	3,832	7,348	70 (3.0)	176 (4.6)	176 (2.4)	3.0
Brazil (PW+)	1,390	1,948	4,046	28 (3.5)	105 (5.4)	105 (2.6)	3.0
China (PW-)	1,938	4,242	5,293	115 (5.9)	115 (2.7)	42 (0.8)	2.3
France (LUP)	4,425	7,171	8.901	137 (3.1)	137 (1.9)	0 (0)	1.8
India (LLP)	1,045	1,436	2,146	0 (0)	36 (2.5)	36 (1.7)	2.0
Italy (LUP)	4,665	8,045	9,347	169 (3.6)	169 (2.1)	0 (0)	1.7
US Corn Belt							
east, rainfed (L)	5,423	7,783	10,143	118 (2.2)	118 (1.5)	118 (1.2)	1.6
west, irrigated (L)	6,629	9,201	11,773	129 (1.9)	129 (1.4)	129 (1.1)	1.5
west, rainfed (L)	3,607	5,285	6,963	84 (2.3)	84 (1.6)	84 (1.2)	1.7

L: linear; QP: quadratic plateau; PW: piecewise with (+) increasing or (-) decreasing rate after breakpoint year; LUP/LLP: linear with upper/lower plateau; EXP: compound exponential.

* Trend-line yields in 1970, 1990, and 2010 were estimated from the best-fit model, which is indicated in parenthesis for each crop-region.

† Absolute rate of gain was calculated from the first derivative of the best-fit model. In some regions, a PW or LP model indicated different yield gain rates for initial years versus recent years. Dividing the rate of gain by the predicted trend-line yield in a given year provides the relative rate of gain, which is expressed as a percentage and shown in parenthesis.

‡ Calculated compound rate of yield increase for the entire time interval (estimated parameter b of EXP model, which is expressed as a percentage).

Supplementary Table S6 Examination of the number of years needed to identify a statistically significant upper yield plateau in crop-region cases exhibiting yield stagnation.

Crop-region	r^{2} *	y_0 (kg ha^{-1})	x_0	Data-year at which x_0 became statistically significant (Student's *t*-test; P<0.01)
Rice				
China	0.97	6,394	1997	2000
Japan	0.63	6,466	1997	2006
Korea, Rep. of	0.81	6,363	1978	1990
California	0.87	8,822	1992	1997
Wheat				
Denmark	0.84	7,208	1995	2001
France	0.93	7,081	1996	2002
Germany	0.94	7,494	2000	2005
India	0.98	2,727	2000	2006
The Netherlands	0.90	8,445	1993	2001
United Kingdom	0.91	7,805	1996	2001
Southern U.S. Great Plains	0.30	2,005	1983	1995
Maize [b]				
East Africa	0.49	1,390	1984	2001
France	0.91	8,901	2003	2008
Italy	0.96	9,347	1997	2003

y_0: yield plateau level (kg ha^{-1}); x_0: breakpoint year. Analyses were based on 46 years of yield data (1965-2010) except for the USA (*n* = 47, 1965-2011 time period).

* Coefficient of determination (r^2) for the linear with upper plateau model fitted to the entire yield trend.

Supplementary Table S7 Goodness of fit for different statistical models fitted to yield trends of the three major cereals crops in selected regions.

	Statistical model *				
Country/region	L	QP	PW †	LP †	EXP
Rice					
Bangladesh	0.92 (234)		**0.98 (120) [+; 1988]**		0.97 (141)
China	0.93 (337)	0.96 (256)		**0.97 (216) [U; 1997]**	0.88 (431)
India	**0.95 (142)**				0.93 (163)
Indonesia	0.91 (297)	0.97 (172)	**0.98 (130) [-; 1987]**		0.85 (380)
Japan	**0.61 (294)**			**0.63 (290) [U; 1997]**	0.60 (297)
Korea, Rep. of	0.62 (566)	0.79 (434)		**0.81 (412) [U; 1978]**	0.60 (588)
Philippines	**0.95 (166)**				0.93 (205)
Thailand	0.86 (151)		**0.95 (89) [+; 1988]**	.	0.90 (129)
United States:					
California	0.77 (640)	0.84 (550)		**0.87 (490) [U; 1992]**	0.74 (683)
southern	0.87 (371)			**0.93 (269) [L; 1980]**	0.90 (331)
Vietnam	0.94 (292)			**0.99 (134) [L; 1979]**	0.98 (177)
Wheat					
Argentina	**0.80 (232)**				**0.80 (234)**
Australia	**0.23 (330)**				**0.22 (333)**
Canada	0.63 (247)			**0.63 (248) [L; 1980]**	0.64 (242)
China	**0.97 (198)**				0.93 (321)
Denmark	0.78 (555)	0.82 (500)		**0.84 (477) [U; 1995]**	0.74 (593)
France	0.86 (521)	0.91 (415)		**0.93 (387) [U; 1996]**	0.81 (605)
Germany	0.92 (418)	0.93 (397)		**0.94 (365) [U; 2000]**	0.89 (487)
India	0.96 (126)	0.97 (111)		**0.98 (94) [U; 2000]**	0.92 (185)
The Netherlands	0.84 (648)	0.89 (522)		**0.90 (503) [U; 1993]**	0.79 (731)
United Kingdom	0.85 (598)	0.90 (488)		**0.91 (451) [U; 1996]**	0.80 (684)
US Great Plains:					
south	0.24 (291)			**0.35 (273) [U; 1983]**	0.23 (293)
central	**0.37 (346)**			0.35 (355) [U; 1984]	**0.36 (349)**
north	0.47 (318)			**0.50 (312) [L; 1979]**	**0.48 (314)**
Maize					
Africa:					
east	0.44 (133)			**0.49 (128) [U; 1984]**	0.42 (135)
central	0.50 (75)			**0.73 (56) [L; 1989]**	0.53 (73)
west	0.87 (116)			**0.91 (96) [L; 1979]**	0.91 (96)
Argentina	0.87 (614)		**0.91 (512) [+; 1990]**		**0.90 (526)**
Brazil	0.87 (314)		**0.96 (183) [+; 1990]**		0.94 (215)
China	0.94 (306)	0.96 (247)	**0.97 (211) [-; 1993]**		0.89 (433)
France	0.91 (555)			**0.91 (536) [U; 2003]**	0.89 (593)
India	0.85 (157)			**0.90 (134) [L; 1978]**	0.88 (142)
Italy	0.90 (602)	0.96 (393)		**0.96 (380) [U; 1997]**	0.85 (759)
US Corn Belt:					
east, rainfed	**0.82 (774)**				**0.83 (757)**
west, irrigated	**0.93 (491)**				**0.93 (490)**
west, rainfed	**0.64 (879)**				**0.65 (867)**

Note that the coefficient of determination (r^2) and root mean square error (in parenthesis; in kg ha^{-1}) are shown for the different statistical models fitted yield to trends of rice, wheat, and maize in selected regions. Best-fit model(s) identified for each crop and country or region is (are) highlighted in bold (see Supplementary Table S4). L: linear; QP: quadratic plateau; PW: piecewise with (+) increasing or (-) decreasing rate after breakpoint year; LP: linear with upper (U)/lower (L) plateau; EXP: compound exponential. Forty-six years of yield data were used in the regression analyses (1965-2010) for all countries except the USA (n = 47, 1965-2011).

[*] Only models with statistically significant parameters are shown (Student's *t*-test; P<0.01). Note that PW and LP models are mutually exclusive. [†] Breakpoint year is shown in brackets.

Supplementary Table S8 Durbin-Watson statistics and tests for normality and variance homogeneity for all crop-region-model cases.

Country/region	Statistical model †				
	L	QP	PW	LP	EXP
Rice					
Bangladesh	0.24 §		**0.88**		0.61
China	0.20 ‡, §	0.35		**0.46**	0.14 ‡, §
India	**1.94**				1.47
Indonesia	0.11‡	0.31	**0.52**		0.07 ‡
Japan	**1.62**			**1.65**	1.54
Korea, Rep. of	0.64	1.03		**1.12**	0.56 §
Philippines	**0.69**				0.46
Thailand	0.60		**1.57**		0.81
United States:					
California	0.94	1.30		**1.61**	0.83
south-central	0.69			**1.30**	0.86
Vietnam	0.23 §			**0.96** §	0.58
Wheat					
Argentina	**2.00**				**1.96**
Australia	**2.12**				**2.09**
Canada	1.41 ‡			**1.43**	1.47 ‡
China	**0.80**				0.31 ‡
Denmark	1.25	1.51		**1.70**	1.10
France	1.12	1.80		**2.05**	0.84 §
Germany	1.54 §	1.73		**2.04**	1.14 §
India	0.81 §	1.06		**1.44**	0.39 §
The Netherlands	1.15	1.92		**1.95**	0.91
United Kingdom	1.02	1.57		**1.81**	0.79
US Great Plains:					
south	1.57			**1.82**	1.55
central	**1.49**			1.44	**1.47**
north	1.55			**1.64**	**1.59**
Maize					
Africa:					
east	1.55			**1.71**	1.52
central	0.57			**1.03**	0.60
west	0.81			**1.19**	1.16
Argentina	1.53 †		**2.09**		**2.07**
Brazil	0.70		**1.95**		1.33
China	0.89	1.36	**1.93**		0.45
France	1.21			**1.32**	1.07
India	1.54			**2.17**	1.88
Italy	0.49	0.98		**1.20**	0.32 §
US Corn Belt:					
east, rainfed	**2.05** ‡				**2.15** ‡
west, irrigated	**1.80** ‡				**1.82** ‡
west, rainfed	**1.79**				**1.83**

Note that Durbin-Watson statistics (DW) are shown for the different statistical models fitted to yield trends of rice, wheat, and maize in selected regions. Serial correlation is significant when DW<1.25 (*P*<0.01). Note that the best-fit model(s) identified for each crop and country or region is (are) highlighted in bold (see Supplementary Table S4). L: linear; QP: quadratic plateau; PW: piecewise with (+) increasing or (-) decreasing rate after breakpoint year; LUP/LLP: linear with upper/lower plateau; EXP: compound exponential. Total number of Forty-six years of yield data were used in the regression analyses (1965-2010) except for the USA (*n* = 47, 1965-2011).

† Only values for models with statistically significant parameters are shown (Student's *t*-test; *P*<0.01). Note that PW and LP models are mutually exclusive.

‡ Residuals distribution deviates from normality (Shapiro-Wilks test; *P*<0.01).

§ Residuals variance is not homogenous across years (Levene test; *P*<0.01).

Supplementary Table S9 Assessment of data transformation impact on model performance for all crop-region-model cases.

Country/region	Statistical model *				
	L	QP	PW	LP	EXP
Rice					
Bangladesh	λ =-1 (0.97) †	.	**λ=1**		λ=1
China	λ=2 (0.95)	λ=1	λ=1	**λ=1**	λ=0 (0.90)
India	**λ=1**	λ=0 (0.94)	λ =-0.5 (0.94)		λ=1
Indonesia	λ=2 (0.95)	λ=0 (0.98)	**λ=1**	λ=1	λ=2 (0.87)
Japan	**λ=1**			**λ=1**	λ =1
Korea, Rep. of	λ=1	λ=-2 (0.88)	λ=-1 (0.90)	**λ =-1 (0.90)**	λ=0 (0.61)
Philippines	**λ=1**				λ=1
Thailand	λ=-0.5 (0.86)		**λ=1**	λ=1	λ=2 (0.92)
United States:					
California	λ=1	λ=1	.	**λ=1**	λ=-0.5 (0.77)
south-central	λ=1			**λ=1**	λ=2 (0.91)
Vietnam	λ=0 (0.95)			**λ=1**	λ=1
Wheat					
Argentina	**λ=1**	λ=0.5 (0.82)	λ=-1 (0.80)		**λ=1**
Australia	**λ=1**				**λ=1**
Canada	λ=1			**λ=1**	λ=1
China	**λ=1**				λ=1
Denmark	λ=1	λ=1		**λ=1**	λ=0 (0.79)
France	λ=1	λ=1		**λ=1**	λ=0 (0.83)
Germany	λ=1	λ=1		**λ=1**	λ=0 (0.83)
India	λ=1	λ=1		**λ=1**	λ=1
The Netherlands	λ=1	λ=1		**λ=1**	λ=0 (0.81)
United Kingdom	λ=1	λ=1		**λ=1**	λ=1
US Great Plains:					
south	λ=1			**λ=1**	λ=1
central	**λ=1**			λ=1	**λ=1**
north	λ=1			**λ=1**	**λ=1**
Maize					
Africa					
east	λ=1	λ=-2 (0.58)		**λ=-1 (0.57)**	λ=1
central	λ=1			**λ=1**	λ=2 (0.52)
west	λ=1			**λ=1**	λ=1
Argentina	λ=0 (0.90)		**λ=1**	λ=1	**λ=1**
Brazil	λ=-1 (0.93)		**λ=1**	λ=1	λ=1
China	λ=1	λ=1	**λ=1**	λ=1	λ=1
France	λ=1			**λ=1**	λ=1
India	λ=0 (0.86)		λ=1	**λ=1**	λ=1
Italy	λ=2 (0.84)	λ=1	λ=-1 (0.97)	**λ=1**	λ=1
US Corn Belt:					
east, rainfed	**λ=1**				**λ=1**
west, irrigated	**λ=1**				**λ=1**
west, rainfed	**λ=1**				**λ=1**

Note that the best-fit model(s) identified for each crop and country or region is (are) highlighted in bold (see Supplementary Table S5). L: linear; QP: quadratic plateau; PW: piecewise; LP: linear plateau; EXP: compound exponential. Forty-six years of yield data were used in the regression analysis (1965-2010) except for USA (n = 47, 1965-2011).

* Only models with statistically significant parameters are shown (Student's *t*-test; P<0.01).

† Recommended transformation for grain yield according to Box-Cox method. If $\lambda = 1$, no transformation is needed; if $\lambda \neq 1$, model performance improves with transformed data (re-calculated r^2 based on transformed grain yield data is shown in parenthesis). Recommended transformation of dependent variable is square, square-root, natural log, inverse square-root, reciprocal, and inverse square for λ = 2, 0.5, 0, -0.5, -1, and -2, respectively.
